# Editorial Note

**Published:** 1917-12

**Authors:** 


### The Medical Bulletin

N° 2 PARIS DECEMBER 1917

### EDITORIAL NOTE

The Medical Bulletin, of which this number is the second issue, will be published about the 15 th of each month. It will be distributed without cost to medical officers in the Allied Armies and to others engaged in war relief work who may find it useful. Several copies will be sent to each hospital formation and individual names will be placed on the mailing list on receipt of a written request sent to the office of the journal.

It will be the object of the Medical Bulletin to present in condensed form the most important developments in war medicine and surgery. Abstracts will be made of articles appearing in European and American periodicals, to the end of making immediately and generally available a knowledge of current work.

The Medical Bulletin will not always confine itself to abstracts from periodicals for the current month, but, in order to present a special problem with more completeness, will occasionally review earlier work that has contributed to the development of the subject. In this issue, for example, an effort has been made to review the most significant work that has been done on Gas Gangrene and Trench Fever.

It is evidently impossible in a journal of this scope to offer a complete bibliography of any subject. Papers will be selected with a view to giving an impartial presentation of various and sometimes divergent opinions, and they will be offered without editorial criticism.

